# The effect of oocyte denudation time and intracytoplasmic sperm injection time on embryo quality at assisted reproductive technology clinic – A cross-sectional study

**DOI:** 10.1016/j.amsu.2022.104234

**Published:** 2022-08-06

**Authors:** Dian Tjahyadi, Hadi Susiarno, Bondan Aria Chandra, Wiryawan Permadi, Tono Djuwantono, Budi Wiweko

**Affiliations:** a Department of Obstetrics and Gynecology Faculty of Medicine Padjadjaran University / Hasan Sadikin Hospital Bandung, Indonesia; b Department of Obstetrics and Gynecology Faculty of Medicine Universitas Indonesia, Indonesia

**Keywords:** Infertility, Assisted reproductive technology, Embrio quality, Oosit denudation, Intracytoplasmic sperm injection

## Abstract

**Background:**

This study aims to determine whether there is an effect of oocyte denudation time and ICSI time on embryo quality at assisted reproductive Technology clinic.

**Methods:**

An observational analytic retrospective study was conduct using cross-sectional study. The subject were oocytes from *in-vitro* fertilization procedures using the ICSI method at the assisted reproductive technology clinic in a private hospital in Bandung for the period 2017 - 2019. Three variables were oocyte denudation time, ICSI time and embryo quality collected from samples that met the research criteria. Data will be grouped based on denudation time (T_1_) and Intracytoplasmic Sperm Injection time (T_2_).

**Result:**

From the 5 groups of denudation time; 3–4 hours, 4–5 hours, 5–6 hours, 6–7 hours and more than 7 hours group, the denudation time of 3–4 hours group showed the highest number (66.7%) for excellent embryo quality results, while denudation time of more than 7 hours showed the lowest number (29.2%) for excellent embryo quality results with p < 0.001. From these figures, it shows that the ICSI time of 3–4 hours is superior to the ICSI time of 2–3 hours because the outcome of excellent embryo quality is higher while the outcome of poor embryo quality is lower, although the difference is not significant (p = 0.140).

**Conclusion:**

This study shows there was a significant difference in the effect of oocyte denudation time on embryo quality at assisted reproductive technology clinic. There was no significant difference in the effect of intracytoplasmic sperm injection (ICSI) time on embryo quality at assisted reproductive technology clinic.

## Highlights

• Oocyte denudation time is a vital parameter in IVF.
• Oocyte denudation time plays a vital role in improving embryo quality.
• Intracytoplasmic sperm injection may not exert a significant role on embryo quality.

## 1 Introduction

Assisted Reproductive Technology (ART) is one technique to deal with infertility, defined as inability to conceive for couples who have had one year of routine sexual intercourse without contraception [1]. *In-vitro* fertilization (IVF) is an assisted reproduction technology in which ovum cells will be fertilized by sperm in an artificial environment. The application of IVF technology is indicated not only in couples with infertility problems due to disorders of the female reproductive organs, but also in couples with oligospermia, asthenospermia, teratospermia and other sperm problems through integration with the Intracytoplasmic Sperm Injection (ICSI) [2]. ICSI is the process of directly injecting a sperm into the ovum as a substitute for the natural fertilization process [3].

The IVF success rate is measured based on the implantation rate (IR) and the pregnancy rate (PR). Based on several studies, one of the key factors that influenced this number was the quality of the embryos which was influenced by the time of oocyte denudation before ICSI was carried out [4].

Several previous studies that have been conducted for studying the duration of oocyte denudation time and ICSI time for optimum embryo quality have not yet been performed involving Indonesian patients. There might be specific demographic differences concerning the Indonesian IVF patients warranting particular therapeutic considerations. Thus, the authors are interested to conduct study about the effect of oocyte denudation time and ICSI time on embryo quality at assisted reproductive technology clinics in Indonesia.

## 2 Methods

This observational analytic retrospective study was conducted using cross-sectional study. The independent variables in this study were oocyte denudation time and time of ICSI and the dependent variable in this study is the quality of the embryo. The instrument used in this study was a data collection table obtained from medical record data of patients who had underwent *in-vitro* fertilization procedures with ICSI at the Assisted Reproductive Technology (ART) Clinic in a private hospital in the period 2017 - 2019. We included only oocytes collected from IVF procedures incorporating ICSI and we excluded oocytes coming from patients >40 years old, oocytes from patients with primary infertility for more than 5 years, endometriosis and polycystic ovarian syndrome (PCOS). The methodology of this study has been constructed in reference to the STROCSS checklist [5].

For this study, ovarian stimulation was performed using standardized protocols of our facility with GnRH. Oocyte was then collected between 33 and 36 hours after hCG administration. Oocytes were then collected and classified according to the corona-cumulus complex or the germinal vesicle appearance [6].

Oocyte denudation was then performed using hyaluronidase, performed between 37 and 40 hours after hCG administration and before insemination. The denudation process was performed at least twice by passing through the oocytes through at least 2 different sized pipettes. The oocytes were then examined under the microscope to observe its integrity and maturation stage.

Sperm collection was performed before oocyte collection, ideally after 3–5 days of no sexual intercourse. The sperm sample was centrifuged, incubated and processed for ICSI.

ICSI is the injection of a sperm into an oocyte. After ICSI, oocyte is examined 12–17 hours later to evaluate for fertilization. Fertilization is confirmed by the presence of 2 pronuclear and 2 polar bodies [7,8].

From the collected data, the data were grouped and analyzed descriptively and analytically. Data were divided into 5 groups of denudation time (T_1_) and 2 groups of Intracytoplasmic Sperm Injection time (T_2_). Group A_1_ were patients with T_1_ 3–4 hours and T_2_ 2–3 hours, Group A_2_ were patients with T_1_ 3–4 hours and T_2_ 3–4 hours, group B_1_ were patients with T_1_ 4–5 hours and T_2_ 2–3 hours, group B_2_ were patients with T_1_ 4–5 hours and T_2_ 3–4 hours, group C_1_ were patients with T_1_ 5–6 hours and T_2_ 2–3 hours, group C_2_ were patients with T_1_ 5–6 hours and T_2_ 3–4 hours, group D_1_ was patients with T_1_ 6–7 hours and T_2_ 2–3 hours, group D_2_ was patients with T_1_ 6–7 hours and T_2_ 3–4 hours, group E_1_ was patients with T_1_ more than 7 hours and T_2_ 2–3 hours. hours, group E_2_ were patients with T_1_ more than 7 hours and T_2_ 3–4 hours. For this study, we had aimed to recruit 210 samples using a significance value alpha of 5% and a 90% study power and by accounting a 10% drop-out rate.

The oocyte denudation time and ICSI time will be presented as means and standard deviation if they are parametric, or median, minimum and maximum values if they are not parametric. Measurement of the effect of oocyte denudation time and ICSI time on embryo quality was performed using SPSS 20.0 with Chi-Square analysis. The test result is statistically significant if the p value is < 0.05.

Ethical approval was granted by the institutional review board and the Research Ethics Committee of Universitas Padjadjaran. There is no patient involvement in this study as this gathered data from patient case notes only. The study's registration in Clinicaltrials.gov is NCT05448859 (URL: https://clinicaltrials.gov/ct2/show/NCT05448859).

## 3 Result and discussion

In Table 1, from the 5 groups of denudation time; 3–4 hours, 4–5 hours, 5–6 hours, 6–7 hours and more than 7 hours group, the denudation time of 3–4 hours group showed the highest number (66.7%) for excellent embryo quality results, while denudation time of more than 7 hours showed the lowest number (29.2%) for excellent embryo quality results. These results are in accordance with the previous study conducted by Kakade S. et al. which shows that a short preincubation time increase the successful rate of fertilization, where the optimal time is 3–5 hours of the preincubation period [9]. This result also answers the question of denudation time which is likely to give the best result or outcome. The length of time before the denudation process is related to the length of time of oocyte incubation before the denudation process which give oocyte time for its maturation and will affect the outcome [9].

**Table 1:** Difference of denudation time on embryo quality.

Denudation time	Embryo Quality (%)				Total
	Excellent	Good	Moderate	Poor	
**3 – 4 hour**	28 (66,7)	4 (9,5)	7 (16,7)	4 (7,1)	**42**
**4 – 5 hour**	31 (58,5)	17 (32,1)	5 (9,4)	0 (0,0)	**53**
**5 – 6 hour**	21 (42,0)	8 (16,0)	16 (32,0)	5 (10,0)	**50**
**6 – 7 hour**	30 (65,2)	11 (23,9)	0 (0,0)	5 (10,9)	**46**
**More than 7 hour**	14 (29,2)	11 (22,9)	23 (47,9)	0 (0,00)	**48**
**Total (%)**	**124 (51,9)**	**51 (21,3)**	**51 (21,3)**	**13 (5,4)**	**239 (100)**

The result of the statistical test of the difference between denudation time and embryo quality showed that the significance value or p was <0.001. Therefore, there is a significant difference between denudation time and embryo quality.

Meanwhile, in Table 2, the results of the excellent embryo quality based on the ICSI time group were found to be lower in the 2 - 3 hours ICSI time group (51.4%), with the poor embryo quality outcomes higher in this time group (9%) compared to the 3 - 4 hours ICSI time group which has excellent embryo quality higher (52.3%) with the poor embryo quality outcome lower (2.3%). From these figures, it shows that the ICSI time of 3–4 hours is superior to the ICSI time of 2–3 hours because the outcome of excellent embryo quality is higher while the outcome of poor embryo quality is lower, although the difference is not significant (p = 0.140).

**Table 2:** Difference of ICSI time on embryo quality.

ICSI Time	Embryo Quality (%)				Total
	Excellent	Good	Moderate	Poor	
**2 - 3 hours**	57 (51,4)	23 (20,7)	21 (18,9)	10 (9,0)	**111**
**3 - 4 hours**	67 (52,3)	28 (29,1)	30 (23,4)	3 (2,3)	**128**
**Total (%)**	**124 (51,9)**	**51 (21,3)**	**51 (21,3)**	**13 (5,4)**	**239 (100)**

This result is in line with the study done by Isiklar A. et al. (2004) which shows a higher fertilization rate, embryo quality and pregnancy rate in the group that received a preincubation period of 2–4 hours before the ICSI procedure [10]. Thus, the ICSI time groups of 2–3 hours and 3–4 hours will have almost the same embryo quality outcome.

## 4 Conclusion

This study shows there was a significant difference in the effect of oocyte denudation time on embryo quality at assisted reproductive technology clinic. On the other hand, there was no significant difference in the effect of intracytoplasmic sperm injection (ICSI) time on embryo quality at assisted reproductive technology clinic.

## Key messages

Oocyte denudation time plays a vital role in improving embryo quality.

## Provenance and peer review

Not commissioned, externally peer-reviewed.

## Author contributions

DT, HS and BAC conceived the study. DT and BAC collected patient data. DT, HS and BAC performed the statistical analysis and reviewed the results. All authors (DT, HS, BAC, WP, TD and BW) participated in the drafting and the approval of the final manuscript version.

## Data availability statement

Anonymised patient data are available upon reasonable written request.

## Funding statement

We do not receive any external funding for this research.

## Ethics approval statement

The institutional review board and the ethical review board of Universitas Padjadjaran exempted this study from an ethics approval.

## Patient consent statement

The patients have consented to the publication of the clinical data with anonymity.

## Permission to reproduce material from other sources

We do not reproduce materials from other sources for this article.

## Research registration

Not applicable.

## Guarantor

The guarantor of this research is Dian Tjahyadi, M.D.

## Declaration of competing interest

The authors declare that we do not have any conflicts of interest.
